# Effects of Visual and Auditory Feedback on the Mixed-Nerve Silent Period During Isometric Knee Extension Torque Control in Female University Basketball Players

**DOI:** 10.7759/cureus.112086

**Published:** 2026-07-05

**Authors:** Shinichi Daikuya, Yumi Okayama

**Affiliations:** 1 Health and Medical Sciences, Hokuriku University, Kanazawa, JPN

**Keywords:** auditory feedback, electromyography, female basketball player, isometric force control, silent period, visual feedback

## Abstract

Sensory feedback plays an important role in isometric force regulation by modulating neural control during voluntary contraction. The mixed-nerve silent period (MnSP) is an electrophysiological index reflecting inhibitory neural mechanisms during voluntary muscle contraction. This study investigated the effects of visual and auditory feedback on MnSP during an isometric knee extension torque maintenance task in trained athletes. Twelve female collegiate basketball players performed a sustained knee extension torque task at 25% of maximal voluntary isometric contraction. The task was performed under two feedback conditions: visual feedback, in which participants adjusted torque by monitoring a real-time display, and auditory feedback, in which visual input was removed and torque was adjusted using standardized verbal instructions. MnSP was recorded from the ipsilateral opponens pollicis muscle following supramaximal median nerve stimulation. MnSP duration did not differ significantly between visual and auditory feedback in either the dominant or nondominant leg. Furthermore, no significant differences were observed between the dominant and nondominant legs within either feedback condition, although a moderate effect size was observed in the nondominant leg. These findings suggest that trained female basketball players exhibit similar MnSP responses under visual and auditory feedback during isometric force regulation.

## Introduction

Force regulation during isometric contraction is achieved by integrating multiple sensory feedback sources, including visual, proprioceptive, and auditory feedback. In particular, visual feedback facilitates the detection and correction of output errors and strongly influences the variability and temporal structure of muscle output [1-4]. The effect of visual feedback also varies between tasks involving force production or pushing against resistance and those that require maintaining a constant level of muscle output [4]. Moreover, during postactivation potentiation, visual feedback may prevent excessive torque production while reducing motor unit firing rates and subjective exertion, thereby modulating neuromuscular output through reduced voluntary drive rather than through enhanced muscle force per se [5]. Furthermore, visual feedback can directly enhance maximal voluntary contraction and power output [6, 7], whereas verbal instruction-based auditory feedback primarily boosts task performance through motivational mechanisms, with relatively limited influence on precise force regulation or maximal output [8-11]. Differences in feedback modality also affect motor unit synchrony. In particular, coherence is higher under auditory feedback, indicating distinct neurophysiological characteristics associated with each modality [12].

Thus, feedback modality not only influences performance outcomes but also the balance between inhibition and excitation within the central nervous system (CNS). The mixed nerve silent period (MnSP) refers to the transient suppression of muscle activity caused by mixed-nerve stimulation during voluntary contraction. MnSP may indicate a combination of subcortical inhibitory mechanisms, including antidromic collision, Renshaw cell inhibition, and high-threshold afferent input [13-15]. The duration of MnSP varies with the contraction intensity [16, 17]. Taken together, these findings suggest that isometric torque maintenance tasks, which impose a high afferent processing load, can appropriately evaluate changes in central inhibitory mechanisms using MnSP. Visual feedback also modulates the long-latency reflex, with increases in the M2 and M3 components observed under predictable stimulation conditions [18, 19]. Recent studies further propose that differences in visuomotor context and the reliability of visual feedback contribute to gain modulation in higher CNS function. State estimation in the motor cortex and parietal association areas have been proposed as underlying mechanisms [20, 21]. Collectively, these findings indicate that the different feedback modalities exert unique effects on motor control mechanisms at multiple levels, from the spinal cord to the cerebral cortex. In relatively automated postural control tasks, such as quiet standing, changes in the visual environment or base of support minimally influence MnSP. In contrast, case studies of patients following ACL reconstruction have reported marked changes in MnSP during the motor reacquisition process, highlighting its clinical relevance as an index of CNS reorganization. Taken together, these findings strongly indicate that different feedback modalities exert distinct effects on the central inhibition-excitation balance during isometric force regulation. However, few studies have directly compared the effects of visual feedback and auditory (verbal instruction) feedback on MnSP. Specifically, only one study has conducted a precise force control task involving a large muscle group, such as knee extension torque maintenance, in healthy individuals without regular sports participation [22]. Furthermore, this study found no significant differences in MnSP in the dominant leg (measured from the ipsilateral opponens pollicis muscle during constant knee extension torque maintenance) between visual and verbal feedback. However, in the nondominant leg, CNS excitability was significantly shortened under verbal instruction feedback [22]. This finding indicates that, in nonathletes, force regulation in the nondominant leg is less refined in nonathletes. Thus, verbal feedback increases the difficulty of fine output adjustment, thereby enhancing CNS excitability.

Therefore, the present study aimed to compare MnSP responses under visual and auditory feedback during an isometric knee extension torque maintenance task in trained female basketball players. We hypothesized that MnSP would not differ between feedback modalities in trained athletes; thus, MnSP would not differ between visual and auditory feedback. This investigation elucidated the regulatory processes of central inhibitory mechanisms during isometric force control, offering fundamental insights into optimizing feedback strategies in motor training and rehabilitation.

## Materials and methods

Twelve female university basketball players were recruited for the study. An a priori power analysis was conducted using SPSS Statistics (version 31.0.0.0, IBM Corp., Armonk, NY, USA). Based on an expected mean difference of 8 ms in MnSP between conditions, assumed standard deviations of 7 ms and 5 ms, a two-sided significance level (α) of 0.05, and a desired statistical power of 0.80, the required sample size was estimated to be 11 participants. The assumptions were based on MnSP values observed in our previous study using a comparable experimental protocol. This study was approved by the Hokuriku University Research Ethics Review Committee for Human Subjects (Approval No. 2023-16, 27/June/2023). All procedures were conducted following the Declaration of Helsinki. Before the experiment and guided by the decision of the Hokuriku University Research Ethics Review Committee for Human Subjects, we informed the participants about the experiment’s details, potential risks, and intentions to publish and subsequently obtained their written informed consent.

**Figure 1:**
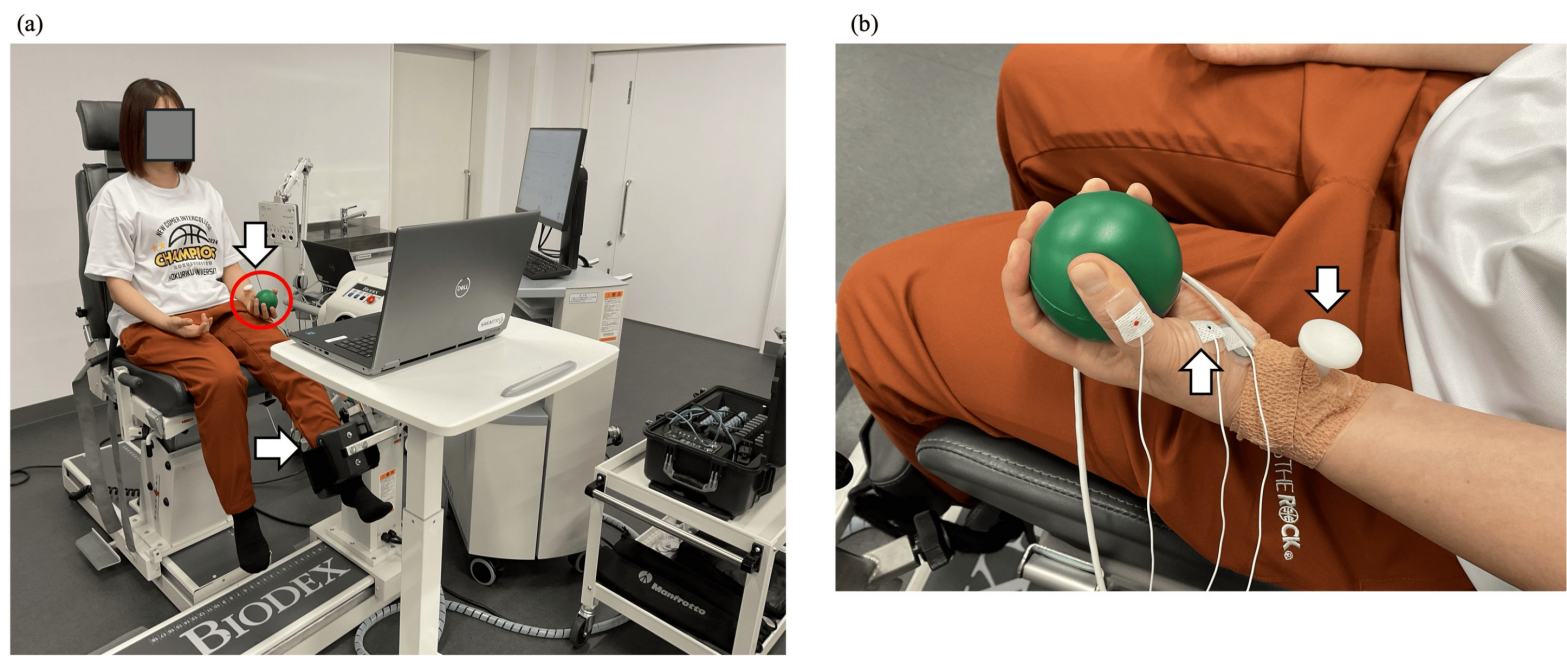
Experiment setup a) Experiment configuration for isometric knee extension synchronized with mixed nerve silent period (MnSP) recording. Participants performed isometric knee extension at 25% of maximal voluntary isometric contraction with the knee flexed to 60 degrees. MnSP was recorded from the opponens pollicis muscle while participants lightly gripped a rubber ball. Arrows indicate the knee extension task and hand position used to maintain slight contraction of the opponens pollicis muscle. (b) Enlarged view of the MnSP recording site. Arrows indicate the stimulation electrode positioned over the median nerve at the wrist and the recording electrode placed over the opponens pollicis muscle. The red circle in panel (a) indicates the area enlarged in panel (b). During the auditory feedback condition, participants performed the task with their eyes closed to prevent visual monitoring of torque output.

The muscle output adjustment task involved maintaining a constant knee extension torque and was performed one side at a time. The muscle output adjustment task was performed using the BIODEX System 4 BDX-4X (Biodex Medical Systems Inc., Shirley, NY, USA). Participants were seated with their knees flexed at 60 degrees. They were then instructed to maintain voluntary isometric contraction of knee extension at 25% of their maximum effort for 60 seconds. The BIODEX system was synchronized with the electromyography measurement system Ultium EMG EM-U880 (Noraxon, Scottsdale, AZ, USA). The torque data from the BIODEX system were imported into the Ultium EMG and displayed on its monitor (Figure 1).

Lower limb laterality was determined as the side with which the participant could more easily kick a ball and was designated as the dominant leg. In the visual feedback (monitor display), participants adjusted their output by observing their own torque curve displayed in real time on a computer monitor. In the auditory feedback (standardized verbal instructions), visual input was removed, and torque output was adjusted based on verbal feedback provided by the same examiner who monitored the torque curve throughout the task. The order of testing for dominant and nondominant legs and for visual and auditory feedback was randomly assigned. The order of the visual and auditory feedback conditions was randomized using a computer-generated randomization sequence.

The MnSP duration was defined as the interval from the onset of the stimulus artifact to the resumption of muscle activity. MnSP during the muscle output adjustment task was recorded from the ipsilateral opponens pollicis muscle using a Neuropack system (Nihon Kohden, Tokyo, Japan), with signals band-pass filtered between 20 and 2,000 Hz. Electromyographic signals were recorded using disposable Ag/AgCl adhesive gel electrodes (NM-317Y3, Nihon Kohden, Tokyo, Japan). Prior to electrode placement, the skin was carefully prepared by abrasion with conductive gel and cleansing with isopropyl alcohol. The stimulation conditions for MnSP measurement were as follows: nerve: median nerve; stimulation site: center of the wrist; intensity: supramaximal stimulation at 120% of the intensity required to elicit the maximal M-wave; frequency: 0.5 Hz; pulse duration: 0.2 ms; and number of stimuli: 16. The recording conditions were as follows: sweep time: 200 ms; muscle: opponens pollicis; side: ipsilateral to knee extension; contraction intensity: light contraction achieved by gently gripping a rubber ball; active electrode: placed over the belly of the opponens pollicis muscle; and reference electrode: placed at the distal end of the proximal phalanx of the thumb. Background electromyographic activity was recorded to ensure consistent contraction of the opponens pollicis muscle across conditions. The MnSP duration was measured for each of the 16 stimuli, and the average value was used as the representative MnSP measurement for each condition. Muscle activity resumption was operationally defined as the first negative deflection exceeding 2 SD below the mean prestimulus baseline electromyographic activity, as calculated from a prestimulus baseline period.

Based on the obtained results, MnSP was compared as follows: (1) between visual and auditory feedback in the dominant leg; (2) between visual and auditory feedback in the nondominant leg; (3) between the dominant and nondominant legs under the visual feedback; and (4) between the dominant and nondominant legs under the auditory feedback.

Statistical analyses were conducted using SPSS (version 31.0.0.0, IBM, Armonk, NY, USA). Normality assessments using the Shapiro-Wilk test and visual inspection of data distributions revealed the non-normal distribution of data. Therefore, values were expressed as medians and interquartile ranges (IQRs). The Wilcoxon signed-rank test was used to compare MnSP between dominant and nondominant legs within each feedback, as well as between visual and auditory feedback within each leg. The effect size (r) was calculated using the following equation:

$$r = \frac{|Z|}{\sqrt{N}}$$

where Z is the standardized test statistic obtained from the Wilcoxon signed-rank test, and N is the total number of observations.

Four pairwise comparisons were specified a priori based on the study hypotheses; therefore, no adjustment for multiple comparisons was applied because these analyses were confirmatory rather than exploratory. The level of statistical significance was set at p < 0.05.

## Results

The participants' mean age was 19.6 ± 1.2 years (range, 18-22 years), and their mean height was 164.8 ± 5.5 cm (range, 156-174 cm). The number of valid observations was 12 for all variables involved in the visual and auditory feedback for both the dominant and nondominant legs. No missing data were identified. Normality assessment indicated non-normal distributions across all conditions (Shapiro-Wilk test); therefore, nonparametric statistics were applied. Median MnSP values are summarized in Table 1.

**Table 1:** MnSP comparison between visual and auditory feedback for dominant and non-dominant legs MnSP - mixed-nerve silent period

Side	Visual / auditory	Median MnSP (IQR) (ms)	Z value	p-value	Effect size (r)
Dominant leg	Visual	110.6 (100.8–113.1)	0.078	0.937	0.02
	Auditory	105.2 (99.3–117.1)			
Non-dominant leg	Visual	105.1 (100.2–110.9)	-1.569	0.117	0.45
	Auditory	102.6 (96.7–109.3)			

Under visual feedback in the dominant leg, the median MnSP was 110.6 (IQR, 100.8-113.1). The distribution exhibited a skewness of 1.36 and a kurtosis of 3.35, indicating a slightly positively skewed and peaked distribution. Under auditory feedback in the dominant leg, the median was 105.2 (IQR, 99.3-117.1). Under visual feedback in the nondominant leg, the median MnSP was 105.1 (IQR, 100.2-110.9). The skewness was 0.41, and the kurtosis was −0.47, indicating no marked deviation from symmetry. Under the auditory feedback in the nondominant leg, the median was 102.6, and the IQR was 96.7-109.3. The skewness was 0.16, and the kurtosis was −0.94, suggesting an approximately symmetrical distribution. No significant differences in MnSP were found between visual and auditory feedback in the dominant leg (Z = 0.078, p = 0.937). The distribution of median differences showed similar numbers of positive and negative values, with no consistent directional trend across conditions. Similarly, MnSP did not differ significantly between feedback in the nondominant leg (Z = −1.569, p = 0.117), although slightly more cases exhibited lower MnSP values under the auditory feedback (Table 1).

Furthermore, no differences were observed between the dominant and nondominant legs within the same feedback (visual: Z = −1.569, p = 0.117; auditory: Z = −1.726, p = 0.084) (Table 2).

**Table 2:** MnSP comparison between dominant and non-dominant legs under the same feedback modality MnSP - mixed-nerve silent period

Feedback modality	Side	Median MnSP (IQR) (ms)	Z value	p-value	Effect size (r)
Visual	Dominant leg	110.6 (100.8–113.1)	−1.569	0.117	0.45
	Non-dominant leg	105.1 (100.2–110.9)			
Auditory	Dominant leg	105.2 (99.3–117.1)	−1.726	0.084	0.50
	Non-dominant leg	102.6 (96.7–109.3)			

## Discussion

In the present study, we investigated the effects of different feedback modalities on MnSP during an isometric knee extension torque maintenance task performed by trained athletes. We found no significant differences in MnSP between visual and verbal (auditory) feedback in either the dominant or nondominant leg. MnSP is considered an electrophysiological index reflecting the integrated activity of multiple inhibitory neural mechanisms involving peripheral, spinal, subcortical, and cortical pathways during voluntary contraction, rather than a direct measure of overall CNS excitability. Therefore, changes in MnSP duration should be interpreted as reflecting modulation of the neural pathways contributing to the silent period rather than changes in global central nervous system excitability. A shortened MnSP duration has been associated with increased CNS excitability [13, 23]. Previous studies have found that increased task difficulty or restrictions on sensory feedback modulate MnSP as well as the long-latency reflex [24-26]. In studies involving healthy adults without regular sports participation, including our previous work, the increase in CNS excitability occurs particularly in the nondominant leg when torque adjustment was performed under verbal instruction without visual confirmation [22]. One possible explanation is that the absence of visual feedback increases uncertainty during motor control, resulting in greater reliance on somatosensory and auditory inputs. This shift in sensory processing may modulate the neural mechanisms contributing to MnSP, thereby influencing silent period duration.

However, despite implementing a similar task structure and feedback, the present study did not demonstrate evident MnSP shortening under auditory feedback in trained athletes. This discrepancy may be attributed to differences in motor experience and participant skill level. Athletes with long-term engagement in competitive sports may exhibit highly optimized sensory integration and motor control during task execution, resulting in reduced dependence on external feedback [27-29]. Accordingly, the absence of MnSP shortening under auditory feedback in the present cohort suggests possible attenuation of the influence of task difficulty on CNS excitability through training. Although no significant differences were observed, a moderate effect size was detected in the nondominant leg during between-condition comparisons. Previous studies have shown that motor control of the nondominant limb requires greater cortical involvement than that of the dominant limb [30], which is supported by the present findings. However, the magnitude of change was smaller than that reported in nonathlete populations, indicating the presence of some degree of neural adaptation, even in the nondominant leg of trained athletes. The comparable MnSP across feedback modalities observed in the present study may therefore reflect a stabilized central motor control strategy developed through long-term training. Therefore, MnSP may not simply be a marker that responds to task difficulty or sensory constraints but rather may reflect the balance between inhibition and excitation shaped by long-term motor experience. The contrast between the present findings and previously reported MnSP shortening in nonathletes confirms the utility of MnSP as a sensitive index of skill-dependent neural adaptation.

Several limitations of this study should be acknowledged. Although MnSP provides useful information regarding neural control during voluntary contraction, it represents the integrated influence of multiple inhibitory mechanisms at peripheral, spinal, subcortical, and cortical levels and therefore cannot identify the specific neural substrate responsible for the observed changes. Furthermore, although the target sample size determined by the a priori power analysis was achieved, the relatively small sample size may have limited the detection of subtle effects. Furthermore, because the participants were limited to female university basketball players and no non-athlete comparison group was included, the generalizability of these findings and the interpretation of possible training-related adaptations remain limited. Future studies must evaluate MnSP measurements with cortical indices, such as transcranial magnetic stimulation (TMS) or simultaneous assessment of LLR, to further elucidate the hierarchical characteristics of neural adaptation in trained athletes.

## Conclusions

In conclusion, the present study was consistent with the finding that differences between visual and auditory feedback during an isometric knee extension torque maintenance task did not significantly affect MnSP in trained athletes. The present findings suggest that trained female basketball players exhibit similar MnSP responses under visual and auditory feedback during isometric force control. Although these findings differ from previous observations in nonathlete populations, the influence of long-term sports training cannot be determined directly because no untrained comparison group was included. Collectively, these results suggest that MnSP reflects not only task characteristics but also motor skill level and extent of neural adaptation. Further investigations incorporating cortical measures, such as TMS, are required to clarify these mechanisms.
